# Effects of physical activity and stress on the relationship between social capital and quality of life among breast cancer survivors

**DOI:** 10.1038/s41598-020-74706-5

**Published:** 2020-10-20

**Authors:** Jie Zhao, Yong Ma, Tetsuya Tanimoto, Akihiko Ozaki, Wan-Li Chen, Jing-Ya Wang, Yu-Xin Zhang, Lin-Li Chen, Ji-Wei Wang, Jin-Ming Yu

**Affiliations:** 1 Key Lab of Health Technology Assessment of Ministry of Health, School of Public Health, Fudan University, 130 Dong-An Road, Shanghai, 200032 China; 2 Xujiahui Street Community Health Service Center, Xuhui District, Shanghai, 200235 China; 3 Medical Governance Research Institute, Tokyo, Japan; 4 Jyoban Hospital of Tokiwa Foundation, Fukuhsima, Japan; 5 Center for Disease Control and Prevention of Minhang District, Shanghai, 201101 China; 6 Minhang Institute, School of Public Health, Fudan University, Shanghai, 201100 China

**Keywords:** Cancer, Oncology

## Abstract

This study aims to investigate the serial multiple mediation of physical activity and perceived stress in the relationship between individual social capital and quality of life (QOL) in breast cancer survivors (BCSs). This study was conducted among 520 BCSs between March and April 2017 in Shanghai, China. Data were collected using the Individual Social Capital Scale, the Health-Promoting Lifestyle Profile-II, the Perceived Stress Scale-14 and the EORTC QLQ-C30. Ordinary least-squares regression and the bootstrap method was used to test the significance of the serial multiple mediation model. The serial-multiple mediations of physical activity and perceived stress were found significant in the relationship of QOL with all five dimensions of individual social capital. The separate mediations of two single mediating variables were found significant in the relationship of QOL with control over life and feeling about the community. In the relationship of QOL with social participation, social network and social support, the separate mediation of physical activity was significant, while the separate mediation of perceived stress was not significant. A multidisciplinary team approach and a variety of delivery systems are needed to address the social, physical and psychological issues for improving QOL among BCSs.

## Introduction

Nowadays breast cancer ranks first in the incidence of female malignant tumors in most countries, including China^1^. With the development of prevention work and advances in diagnosis and treatment technology, an increasing number of breast cancer patients are diagnosed and treated more timely and effectively than before, enjoying better survival prospects. As a result, a higher attention is paid to overall health and quality of life (QOL) of breast cancer survivors (BCSs). The World Health Organization defines QOL as one’s perception of his or her life situation in cultural context and value system^2^, and this measure has been widely used to evaluate the treatment effect and recovery status of cancer survivors^3^.

Definitions of social capital depend partly on the originating discipline, but it is generally regarded as resources that can be accessed from groups or networks that individuals belong to^4^. Social capital is often divided into structural (participation in social activities and support from individuals in the community) and cognitive domains (perceptions of support, reciprocity and trust) based on differences in their health effects^5^. It is further separated to individual and collective social capital. Individual social capital has been broadly defined as the ability of actors to secure benefits by virtue of membership in social networks and other social structures^5^, while collective social capital is viewed as a collective feature and refers to the rules that promote collective action^5^, and the former is reported to exert higher protective health effects than the latter^6^.

Portes^7^ adds to the concept of individual social capital when distinguishing between sources and effects of social capital. He makes a distinction between characteristics of the networks per se (i.e. motivations to make resources available) as the sources, while the actual resources provided (e.g. information, support, and opportunities) are defined as the effects of individual social capital. According to Portes, the consequences of individual social capital include social support, social influence, social control, social participation and material resources^7^. Critics have questioned whether social capital adds anything new to the field of social networks or social support and health, or if it is like ‘pouring old wine into new bottles’^8^. However, the conceptualization of different forms or types of social capital can guide the mapping of the kinds of networks available and for whom, and can guide the evaluation of theory-driven social network interventions. Within this view, social capital has the potential to add new aspects.

In the previous literature, three main pathways linking individual social capital to health outcomes have been reported^9^. Firstly, health resources may be directly related to health. For example, health can be affected by access to relevant resources such as medical care or health insurance through social networks^9^. Secondly, individual social capital could affect health through psycho-social process. For example, social support can act as a buffer, reducing people's stress levels^10^, and social participation can provide people with opportunities to learn new knowledge and skills, and it can also give people a sense of belonging^9^. Thirdly, individual social capital could influence health by the way of health behaviors. For example, social influences, including those of peers, families and social groups, can influence health behaviors such as physical activity, smoking and diet, and thus impact on health outcomes^9^.

Some researchers are changing their views on breast cancer treatment, with a growing emphasis on the role of socio-contextual factors in QOL of BCSs. Individual social capital has been widely concerned in the health-related social context^11^. Among BCSs, it was found that improving individual social capital can enhance cancer treatment compliance^11^, help reduce cancer-related pain^11^, and increase resilience to cancer side effects^12^. Several studies have found that higher levels of social support are significantly associated with higher QOL in BCSs^13,14^. Individual social capital may explain more differences in QOL of BCSs than clinical characteristics^11^. Given the above three widely accepted pathways linking individual social capital to health outcomes, future research could explicitly test these explanations with regard to the QOL among BCSs.

In this context, physical activity as a health behavior-related variable and perceived stress as a psychosocial process-related variable may be important elements which could mediate the association of individual social capital and QOL among BCSs., It has been revealed that individual social capital are significantly associated with physical activity^15^ and perceived stress^16^ among BCSs, and that physical activity^17^ and perceived stress^18^ have been shown to have a significant impact on QOL among BCSs. Furthermore, a few studies have investigated the mediating role of physical activity and perceived stress in the relationship between individual social capital and health outcomes in the general population. A cross-sectional study which used nationally representative data from the 2014 China Family Panel Studies (n = 28,916) shows that physical activity may influence the relationship between self-rated health and social trust and social relationship^19^. Another cross-sectional study which used data from the Health 2000 Survey (n = 8028) of the adult population in Finland found that part of the association between social participation and networks and self-rated health was explained by physical activity^20^. In addition, a cross-sectional study which recruited patients(n = 508) attending a publicly-funded sexually transmitted disease clinic found that perceived stress may mediated the relation between individual-level objective and subjective socioeconomic status and perceived health^21^. However, nowadays few studies have tested the mediation in the relationship of individual social capital with QOL among BCSs.

In this study, physical activity and perceived stress were selected to explore their mediations in the relationship between individual social capital and QOL among BCSs. Since extant literature largely concludes there is reciprocal relationship between physical activity and perceived stress^22^, the serial multiple mediation model was selected for mediation analysis rather than the parallel multiple mediation model, which assumes no relationship between mediation variables. From the perspective of intervention, enhancing physical activity such as yoga and exercise, has been widely proven to be an effective and important way for BCSs to cope with stress^23,24^. Therefore, in this study, physical activity was taken as the first mediating variable and perceived stress as the second mediating variable. We hypothesized that there are three crucial pathways through which individual social capital impacts on QOL: (1) physical activity partly mediated the relationships between individual social capital and QOL; (2) perceived stress partly mediated the relationships between individual social capital and QOL; (3) whether the serial-multiple mediation of physical activity and perceived stress in the relationship between individual social capital and QOL was found to be statistically significant.

## Methods

### Recruitment

Participants of this study were registered members of Shanghai Cancer Rehabilitation Club (SCRC), which has a three-level management network of city, district and street and recruit members from community and hospitals through extensive recruitment channels covering all 17 districts of Shanghai^25^. There is a total of about 50,000 BCSs in Shanghai, and about 5,000 registered BCSs in SCRC. Before the subjects were recruited, we estimated the sample size required for the mediation effect analysis. Using the empirical power tables26, it was shown that for the bias-corrected bootstrap test of mediation, a sample size of 400, or a larger sample if measurement error is present, is required for 0.8 power^26^. Thus, we randomly selected four branches of the SCRC which had a total of about 1000 registered BCSs. We connected the four branches by email or telephone and they agreed to participate in the study. Then we sent recruitment advertisements and posters to them. They disseminated the recruitment information to their members by WeChat and during club activities. From March to April 2017, a total of 532 BCSs were recruited from four districts. Other BCSs in the four districts who were not recruited either did not meet the inclusion criteria, did not value the recruitment information, or did not consider the study to be of tangible benefit to them and did not want to participate. The inclusion criteria for this study including: (1) female and above 16 years old; (2) breast cancer as the first primary cancer and willing to complete treatment; (3) be able to participate in cancer club activities independently; (4) no cognitive impairment. Participants who meet the inclusion criteria will be included in the study.

There is a variety of cancer rehabilitation activities in the club, such as support groups, image guidance, music therapy and cognitive behavioral therapy, which are held regularly once a month. Before or after the activity, participants filled out questionnaires on the spot under the guidance of members of the research team. If any of the participants had difficulty completing the questionnaire, the investigators would read the questions one by one for them and recorded their answers. Before the participants filled out the self-reported questionnaire, they were informed the purpose of the study and that the investigation was anonymous and voluntary. Finally, 520 questionnaires were collected. The response rate was 97.7%, and the valid rate was 100%. The study was approved by the Medical Research Ethics Committee of the School of Public Health, Fudan University (The international registry NO. IRB00002408 & FWA00002399). A written Informed Consent was obtained from each participant prior to participation in the study.

### Measurement

#### Measurement of social capital

Individual social capital was measured in both the cognitive and structural domains by the scale which was developed by the research group and mainly refers to “Social Capital of Organization—World Bank”^27^, “Social Capital of Organization”^28^, “Social Capital and mental health and well-being”^29^. This scale had a 1-month time frame. The scale contains 24 items which were divided into two domains including five dimensions: structural social capital (social participation, social network, social support) and cognitive social capital (control over life and feeling about the community). In this scale, social participation measures participants' participation in social activities, such as volunteer activities, social group activities, community activities or leisure activities. Social network measures how closely participants spend time with friends, relatives and others, and emphasizes the strength of the relationship between the subjects and the people around them. Social support measures whether participants are able to get help and attention from others when they need it, and whether anyone are willing to listen attentively to participants. Control over life measures the degree of self-esteem and confidence of subjects, as well as their life satisfaction. Feeling about the community measures the satisfaction degree of subjects with the overall environment and public facilities of the community they live in, and emphasizes the degree of integration and acceptance of respondents as individuals in the community. All items were standardized to a 5-point Likert scale, ranging from 1(never) to 5 (always). The higher the summary score, the higher the level of social capital. The scale had been found to have good reliability and validity^30,31^. In this study, the Cronbach's α of the scale was 0.950, and the Cronbach's α of social participation, social network, social support, control over life and feeling of community were 0.913, 0.867, 0.860, 0.913 and 0.947, respectively, indicating that the component scales had good internal consistency reliability.

#### Measurement of physical activity

Physical activity was measured by the dimension of physical activity in the health-promoting lifestyle profile-II (HPLP-II). HPLP-II English version was a multi-dimensional scale developed by Walker^32^ to measure people's health-promoting lifestyle. It contains 52 items, which were divided into six dimensions: health responsibility, physical activity, nutrition, mental growth, social contact and stress management. Our team translated the physical activity dimension of HPLP-II into Chinese to measure the level of physical activity. This dimension contained eight items which asked the subjects about the current frequency and intensity of their physical activity and used a four-point response format (“never”, “sometimes”, “often” and “always”). Physical activity scores were obtained by summing the scores across all 8 items. In this study, the Cronbach's α of Chinese version of physical activity dimension was 0.836, indicating that the component scale had good internal consistency reliability.

#### Measurement of perceived stress

Cohen and colleagues developed the original 14-item English version of the Perceived Stress Scale (PSS-14) as a global measure of stress level by asking respondents to report whether their lives seem to be unpredictable, uncontrollable or overloaded^33^. This scale had a 1-month time frame. PSS-14 scores were obtained by reversing the scores on the seven positive items, e.g., 0 = 4, 1 = 3, 2 = 2, etc., and then summing across all 14 items. Items 4, 5, 6, 7, 9, 10, and 13 were the positively stated items. The scale had been translated into several languages, including Spanish, Swedish, Japanese and Chinese. In this study, perceived stress was measured by the Chinese version of the Perceived Stress Scale-14 (PSS-14). The Chinese version of the PSS-14 had also been used in research areas such as mental health and physical activities and had been found to have high internal consistency, construct validity and concurrent validity^34^. In this study, the Cronbach's α of PSS-14 Chinese version was 0.880, indicating that it had good internal consistency reliability.

#### Measurement of QOL

QOL was measured by EORTC QLQ-C30 which was developed by the European Organization for Research and Treatment of Cancer (EORTC). The 30-item EORTC QLQ-C30 version 3.0 consists of five multi-item function scales (physical, role, cognitive, emotional, and social), three multi-item symptom scales (fatigue, nausea and vomiting, and pain), six single-item symptom scales (dyspnea, insomnia, appetite loss, constipation, diarrhea, and financial impact), and a two-item global QOL scale (QL). The questionnaire had a 1-week time frame and used a four-point response format (“not at all”, “a little”, “quite a bit” and “very much”), with the exception of the global QL scale, which has a seven-point response format. According to scoring algorithm for generating the QLQ-C30 summary score developed by N.K. Aaronson^35^, the EORTC QLQ-C30 Summary Score was calculated from the mean of 13 of the 15 QLQ-C30 scales (the Global QOL scale and the Financial Impact scale are not included). Prior to calculating the mean, the symptom scales need to be reversed to obtain a uniform direction of all scales. A higher QLQ-30 Summary Score indicates better QOL. The highest QLQ-30 Summary Score is 100. The Chinese version of EORTC QLQ-C30 scale had also been proved to have good reliability and validity^36^. In this study, the Cronbach's α of EORTC QLQ-C30 Chinese version scale was 0.831, indicating that it had good internal consistency reliability.

### Statistical analyses

Descriptive analysis, independent-samples T-test and one-way analysis of variance (ANOVA) were used to describe and compare the demographic data (age, body mass index [BMI], education, marital status, personal monthly income, duration of disease) and the distribution of QOL. Pearson correlation analyses of the eight variables (five dimensions of social capital, physical activity, perceived stress and QOL) were performed using SPSS version 19.0. To test the significance of the multiple-mediator model, we adopted the model 6 of PROCESS macro for SPSS provided by Hayes^37^. This approach was based on ordinary least-squares regression and the bootstrap method^37^. In this study, 5,000 bootstrap bias-corrected 95% confidence intervals (BC CI) was used for mediation analyses in the test from the Serial-Multiple Mediation Model 6, and if BC CI did not contain zero, the mediation was considered significant^43^. And in this study, non-standardized Beta coefficients are calculated in order to reduce Type 1 errors due to distribution. Furthermore, age, BMI, education, marital status, personal monthly income and duration of disease were treated as covariates.

### Ethics approval

The study was approved by the Medical Research Ethics Committee of the School of Public Health, Fudan University (The international registry NO. IRB00002408 & FWA00002399), and all methods were carried out in accordance with relevant guidelines and regulations.

## Results

### Preliminary analyses

The socio-demographic information for participants and the distribution of QOL were summarized in Table 1. Date from 520 BCSs were collected. The average age of the study population was 61.36 ± 6.72 years (mean ± SD). The average QLQ-C30 summary score was 84.30 ± 10.64. Independent-samples T-test and one-way ANOVA showed there were significant differences in QOL in marital status (F = 6.341, p = 0.002) and personal monthly income (F = 5.788, P = 0.003) but not in age (t = -1.389, p = 0.166), BMI (F = 0.447, P = 0.719), educational attainment (F = 0.583, P = 0.558) and duration of disease (F = 0.646, P = 0.586) among BCSs.

**Table 1:** Descriptive statistics and the distribution of QOL.

Variables	Characteristics	N (%)	QLQ-C30 Summary Score mean (SD)
Age	< 60	190 (36.5)	83.46 (10.70)
	≥ 60	330 (63.5)	84.79 (10.60)
t		− 1.389	
P		0.166	
BMI	< 18.5	14 (2.7)	82.70 (16.13)
	18.5–23.9	272 (52.3)	84.61(9.92)
	24–27.9	195 (37.5)	83.78 (11.55)
	≥ 28	39 (7.5)	85.24 (8.71)
F		0.447	
P		0.719	
Marital status	Single	6 (1.2)	90.60 (7.26)
	Cohabitated or married	460 (88.5)	84.79 (10.32)
	Lived alone (widowed/divorced/separated)	54 (10.3)	80.26 (12.16)
F		6.341	
P		0.002	
Educational attainment	Less than high school	200 (38.5)	83.68 (11.29)
	High school	234 (45.0)	84.60 (10.32)
	Bachelor or higher	86 (16.5)	84.90 (10.04)
F		0.583	
P		0.558	
Personal monthly income (RMB)	< 3000	220 (42.3)	82.55 (10.91)
	3000–5000	283 (54.4)	85.78 (10.36)
	> 5000	17 (3.3)	84.62 (9.23)
F		5.788	
P		0.003	
Duration of disease(years)	0–4	155 (29.8)	83.27 (10.57)
	5–9	160 (30.8)	84.49 (10.17)
	10–19	149 (28.7)	84.41(11.19)
	≥ 20	56 (10.8)	85.49 (11.03)
F		0.646	
P		0.586	

### Preliminary correlation analyses

Table 2 presents the relationship between variables. There is a significant correlation between social capital, physical activity, perceived stress and QOL. The correlation coefficient varied from 0.159 to 0.647.

**Table 2:** Pearson correlation coefficient values regarding study variables.

	1	2	3	4	5	6	7	8
1. Social participation	1							
2. Social networks	0.409**	1						
3. Social support	0.506**	0.519**	1					
4. Control over life	0.431**	0.500**	0.647**	1				
5. Feelings about the community	0.297**	0.344**	0.532**	0.532**	1			
6. Physical activity	0.340**	0.323**	0.327**	0.317**	0.301**	1		
7. Perceived stress	− 0.225**	− 0.206**	− 0.301**	− 0.419**	− 0.319**	− 0.219**	1	
8. QOL	0.161**	0.190**	0.159**	0.207**	0.238**	0.259**	− 0.427**	1

*p < .05; **p < .01.

### Mediation analyses

When the five dimensions of individual social capital – social participation, social networks, social support, control over life, and feelings about the community – were taken as independent variables respectively, the serial multiple mediation analyses of physical activity and perceived stress in the relationship between these independent variables and QOL based on ordinary least-squares regression analysis and bootstrap method were as follows.

#### Social participation as the independent variable

**Figure 1:**
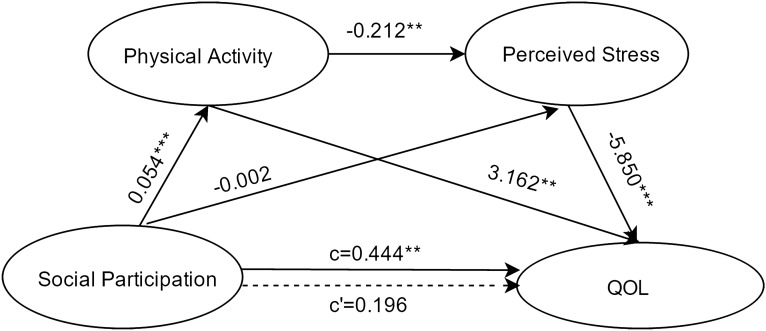
Serial multiple mediation of physical activity and perceived stress in the relationship between social participation and QOL with non-standardized beta values. c is the total effect of social participation on QOL. c' is the direct effect of social participation on QOL, represented by a dotted line. The other values in the figure are regression coefficients in the model. *p < .05, **p < .01, ***p < .001.

Results about the serial-multiple mediation of physical activity and perceived stress in the relationship between social participation and QOL based on ordinary least-squares regression analysis are presented in Fig. 1. The results indicated that the path through both mediators was significant. On the other hand, the path through physical activity alone was also significant, while the path through perceived stress alone was not significant.

Results about the serial-multiple mediation of physical activity and perceived stress in the relationship between social participation and QOL based on bootstrap method are presented in Table 3. The total indirect effect (the difference between total and direct effects/c–c’) of social participation through physical activity and perceived stress on QOL was statistically significant (point estimate = 0.2458; 95%BC CI [0.0873,0.4258]). When considering the mediating variables separately and together, single mediation of physical activity (point estimate =  = 0.1700; 95%BC CI [0.0788,0.2769]), serial-multiple mediation of physical activity and perceived stress (point estimate = 0.0668; 95%BC CI [0.0210,0.1214]) were found statistically significant, while single mediation of perceived stress (point estimate = 0.0116; 95%BC CI [-0.1216,0.1489]) was found to be insignificant.

**Table 3:** The Indirect effects of five dimensions of individual social capital(X) on QOL(Y) through physical activity(M_1_) and perceived stress(M_2_) with non-standardized values.

Model	Path	Point estimate	SE	Bootstrapping 95% BC CI
X_1_ → Y	Total indirect effect	0.2485*	0.0855	(0.0873,0.4258)
	Ind1	0.1700*	0.0504	(0.0788,0.2769)
	Ind2	0.0116	0.0673	(-0.1216,0.1489)
	Ind3	0.0668*	0.0257	(0.0210,0.1214)
X_2_ → Y	Total indirect effect	0.2542*	0.1012	(0.0684,0.4686)
	Ind1	0.1609*	0.0503	(0.0697,0.2679)
	Ind2	0.0275	0.0823	(-0.1262,0.1992)
	Ind3	0.0658*	0.0267	(0.0191,0.1238)
X_3_ → Y	Total indirect effect	0.2872*	0.0847	(0.1350,0.4616)
	Ind1	0.1431*	0.0430	(0.0669,0.2380)
	Ind2	0.0995	0.0702	(-0.0300,0.2454)
	Ind3	0.0446*	0.0194	(0.0081,0.0852)
X_4_ → Y	Total indirect effect	0.6031*	0.1542	(0.3360,0.9437)
	Ind1	0.2270*	0.0711	(0.1051,0.3849)
	Ind2	0.3137*	0.1295	(0.0869,0.5918)
	Ind3	0.0624*	0.0324	(0.0026,0.1315)
X_5_ → Y	Total indirect effect	0.5040*	0.1237	(0.2824,0.7665)
	Ind1	0.1766*	0.0562	(0.0793,0.2988)
	Ind2	0.2771*	0.1030	(0.0895,0.4921)
	Ind3	0.0504*	0.0263	(0.0029,0.1048)

Number of bootstrap samples = 5000. X_1_: Social participation; X_2_: Social networks; X_3_: Social support; X_4_: Control over life; X_5_: Feeling about the community. Ind 1: X → M_1_ → Y; Ind 2: X → M_2_ → Y; Ind 3: X →  M_1_ → M_2_ → Y; * BC CI did not contain zero, the mediation was considered significant, p < 0.05.

BC CI, bias-corrected confidence interval.

#### Social network as the independent variable

**Figure 2:**
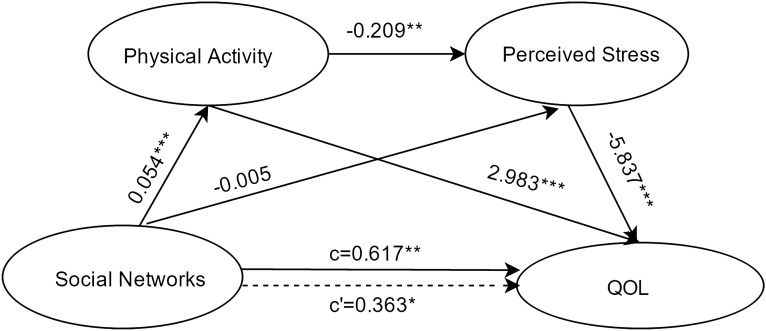
Serial multiple mediation of physical activity and perceived stress in the relationship between social networks and QOL with non-standardized beta values. c is the total effect of social participation on QOL. c' is the direct effect of social participation on QOL, represented by a dotted line. The other values in the figure are regression coefficients in the model. *p < .05, **p < .01, ***p < .001.

Results about the serial-multiple mediation of physical activity and perceived stress in the relationship between social network and QOL based on ordinary least-squares regression analysis are presented in Fig. 2. The results indicated that the path through both mediators was significant. However, the path through physical activity alone was also significant, while the path through perceived stress alone was not significant.

Results about the serial-multiple mediation of physical activity and perceived stress in the relationship between social network and QOL based on bootstrap method are presented in Table 3. The total indirect effect of social network through physical activity and perceived stress on QOL is statistically significant (point estimate = 0.2542; 95%BC CI [0.0684,0.4686]). When considering the mediating variables separately and together, single mediation of physical activity (point estimate = 0.1609; 95%BC CI [0.0697,0.2679]), serial-multiple mediation of physical activity and perceived stress (point estimate = 0.0658; 95%BC CI [0.0191,0.1238]) were found statistically significant, while single mediation of perceived stress (point estimate = 0.0275; 95%BC CI [− 0.1262,0.1992]) was found to be insignificant.

#### Social support as the independent variable

**Figure 3:**
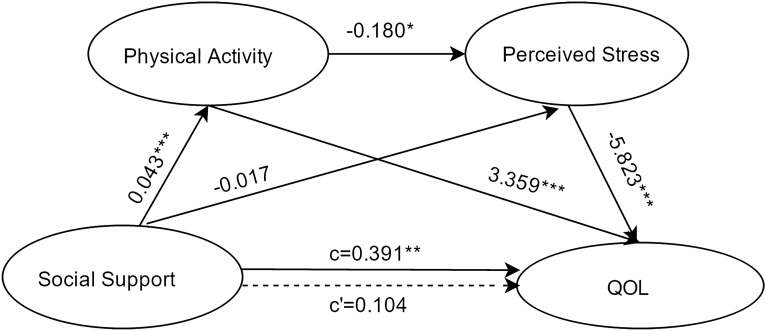
Serial multiple mediation of physical activity and perceived stress in the relationship between social support and QOL with non-standardized beta values. c is the total effect of social participation on QOL. c' is the direct effect of social participation on QOL, represented by a dotted line. The other values in the figure are regression coefficients in the model. *p < .05, **p < .01, ***p < .001.

Results about the serial-multiple mediation of physical activity and perceived stress in the relationship between social support and QOL based on ordinary least-squares regression analysis are presented in Fig. 3. The path through both mediators was significant. On the other hand, the path through physical activity alone was also significant, while the path through perceived stress alone was not significant. The results were similar to those obtained when social participation and social networks were the independent variables.

Results about the serial-multiple mediation of physical activity and perceived stress in the relationship between social support and QOL based on bootstrap method are presented in Table 3. The total indirect effect of social support through physical activity and perceived stress on QOL was statistically significant (point estimate = 0.2872; 95%BC CI [0.1350,0.4616]). When considering the mediating variables separately and together, single mediation of physical activity (point estimate = 0.1431; 95%BC CI [0.0669,0.2380]), serial-multiple mediation of physical activity and perceived stress (point estimate = 0.0446; 95%BC CI [0.0081,0.0852]) were found statistically significant, while single mediation of perceived stress (point estimate = 0.0995; 95%BC CI [− .0300,0.2454]) was found to be insignificant.

#### Control over life as the independent variable

**Figure 4:**
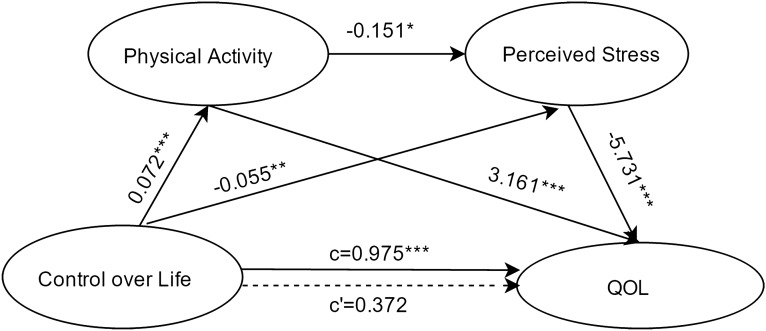
Serial multiple mediation of physical activity and perceived stress in the relationship between control over life and QOL with non-standardized beta values. c is the total effect of social participation on QOL. c' is the direct effect of social participation on QOL, represented by a dotted line. The other values in the figure are regression coefficients in the model. *p < .05, **p < .01, ***p  < .001.

Results about the serial-multiple mediation of physical activity and perceived stress in the relationship between control over life and QOL based on ordinary least-squares regression analysis are presented in Fig. 4. The results indicated that the path through both mediators was significant. Moreover, the path through both physical activity alone and perceived stress alone were also significant.

Results about the serial-multiple mediation of physical activity and perceived stress in the relationship between control over life and QOL based on bootstrap method are presented in Table 3. The total indirect effect of control over life through physical activity and perceived stress on QOL was statistically significant (point estimate = 0.6031; 95%BC CI [0.3360,0.9437]). When considering the mediating variables separately and together, single mediation of physical activity (point estimate = 0.2270; 95%BC CI [0.1051,0.3849]), serial-multiple mediation of physical activity and perceived stress (point estimate = 0.0624; 95%BC CI [0.0026,0.1315]), and single mediation of perceived stress (point estimate = 0.3137; 95%BC CI [0.0869,0.5918]) were found statistically significant.

#### Feeling about the community as the independent variable

**Figure 5:**
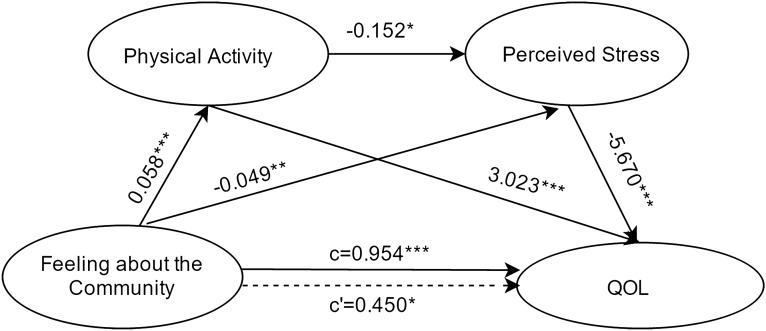
Serial multiple mediation of physical activity and perceived stress in the relationship between feeling about the community and QOL with non-standardized beta values. c is the total effect of social participation on QOL. c' is the direct effect of social participation on QOL, represented by a dotted line. The other values in the figure are regression coefficients in the model. *p < .05, **p < .01, ***p  < .001.

Results about the serial-multiple mediation of physical activity and perceived stress in the relationship between feeling about the community and QOL based on ordinary least-squares regression analysis are presented in Fig. 5. The results indicated that the path through both mediators was significant. Moreover, the path through both physical activity alone and perceived stress alone were also significant. The results are similar to that obtained when control over life is the independent variable.

Results about the serial-multiple mediation of physical activity and perceived stress in the relationship between feeling about the community and QOL based on bootstrap method are presented in Table 3. The total indirect effect of feeling about the community through physical activity and perceived stress on QOL was statistically significant (point estimate = 0.5040; 95%BC CI [0.2824,0.7665]). When considering the mediating variables separately and together, single mediation of physical activity (point estimate = 0.1766; 95%BC CI [0.0793, 0.2988]), serial-multiple mediation of physical activity and perceived stress (point estimate = 0.0504; 95%BC CI [0.0029,0.1048]), and single mediation of perceived stress (point estimate = 0.2771; 95%BC CI [0.0895,0.4921]) were found statistically significant.

## Discussion

The results showed that physical activity and perceived stress could separately mediate the relationships between individual social capital and QOL among BCSs, and besides, the serial-multiple mediations of physical activity and perceived stress in the relationship of QOL with individual social capital was also found to be statistically significant among BCSs, though the extent of the association with the QOL and its mediators would differ between the individual dimensions of social capital.

With regard to our finding that the physical activity mediated the relationships between individual social capital and QOL, numerous studies have revealed a positive relationship between social support and cancer survivors’ physical activity. In addition, it has been demonstrated that cancer survivors with more social networks (i.e., talking to family/friends about health) were more likely to pay attention to new exercise recommendations^38^. Also, the beneficial effects of regular physical activity on QOL in breast cancer survivors have been demonstrated in the previous literature^17^. Furthermore, some cross-sectional mediation studies conducted in various general population found that physical activity mediated the relationship of self-rated health with social participation and networks, social trust and social relationship^19,20^.

In this study, in the relationship of QOL with social participation, social network and social support (all belong to the structural social capital domain), the separate mediation of perceived stress was not at a statistically significant level. One possible explanation is that, physical activity is more likely to attenuate the relationship between structural social capital rather than cognitive social capital and perceived stress. Some studies may support this postulations. In a U.S. residents sample (N = 4601), a study showed that physical activity was more strongly associated with structural social capital (social participation) compared with cognitive social capital(social trust)^39^. Another two population based studies which although only look at structural social capital show that structural social capital(social participation) is the strongest predictor of physical activity among psycho-social variables investigated^40,41^. Therefore, given that the effects of physical activity on perceived stress have been widely demonstrated, the role of structural social capital on perceived stress is likely to be more susceptible to the effects of physical activity than cognitive social capital. A further possible explanation from the perspective of structural and cognitive social capital is that cognitive social capital and perceived stress both involve psychosocial processes, so cognitive social capital may be a more proximal determinant of perceived stress than structural social capital. Some researches seem to support this explanation. A study from Peru, which aimed to evaluate the association between chronic Post-Traumatic Stress Disorder (PTSD) and both structural and cognitive social capital in adult survivors(n = 1012) of the earthquake, found that cognitive social capital, but not structural social capital, has a protective influence on the occurrence of chronic PTSD in survivors of natural disasters^42^. Another study which used a sample of 1586 same-sex twin pairs found that only cognitive social capital rather than structural social capital was significantly associated with less depressive symptoms within-pairs^43^.

In particular, this study also demonstrated that physical activity and perceived stress were the serial-multiple mediators which link all dimensions of individual social capital with QOL among BCSs. This pathway means that positive individual social capital was sequentially associated with increased physical activity first and then decreased perceived stress, which was in turn related to improved QOL of BCSs. This finding is also supported by the existing literatures. On the basis that social capital has been shown to have a significant effect on physical activity, furthermore, there is growing evidence to support the view that increasing physical activity could improve QOL of BCSs by improving their psychosocial processes^44,45^.

The research finding that physical activity and perceived stress played a mediating role in the relationships between individual social capital and QOL among BCSs suggests that if there were constraints which exist among BCSs and restrain the improvement of physical activity and the reduction of stress level, the mediating effects of physical activity and perceived stress may be weaken, and as a result, enhancing social capital may not have a positive impact on the QOL. This suggests that in the intervention practice, the constraints should be identified and eliminated while individual social capital being enhanced, so as to ensure the effectiveness of individual social capital intervention on QOL of BCSs. Besides, the motivators which could promote the improvement of physical activity and the reduction of perceived stress should be strengthened, so as to increase the direct benefits of improving physical activity and reducing the perceived stress on QOL of BCSs.

Many social capital interventions have been developed and applied to the field of health promotion. For example, a home visit programme used social trust and sense of security in the community, two forms of individual social capital, as mediators to reduce stress in mothers of newborns^46^. Some researches have developed social software-based self-help bulletin boards, discussion boards and forums which BCSs can use to exchange information and share their experiences^47,48^. These techniques go beyond emotional support and also include informational and instrumental support from patients who develop expertise as they go through treatment^47,48^. These interventions to change individual social capital are also feasible for Chinese BCSs. Besides, in China, some cancer rehabilitation clubs provide social support through psycho-social interventions such as recreational and sports activities, hospice care and cognitive behavioral therapy^49,50^. These cancer rehabilitation clubs also promote the social participation of cancer survivors and help them build informed social networks for cancer treatment and recovery^49,50^. To effectively strengthen social capital, the interventions should be developed within an ecological framework that considers many levels of influence^51^. Based on social software with good information dissemination function, or community organizations with strong leadership, promoting the effective integration and rational distribution of material capital, human capital and information capital in the field of health promotion, and developing specific intervention activities targeted at the target population are the common strategies to strengthen individual social capital^46–51^.

In China, social roles and function of women are often limited by family, politics and traditional culture factors^52^. For women with breast cancer, the scope of the social network and the level of social participation is more likely to be limited than that of healthy women and men^11^. Combined with the findings of this study, Chinese women with breast cancer should be encouraged to participate more in society and establish and maintain their own social network, so that they can get more social support and improve their QOL. The limitations of social networks and social participation of women seem to be similar across cultures. A cross-cultural study with a sample of 112,000 profile pictures from nine world regions found that women favour dyadic relations, and thus appear more often to focus their social capital on only one person at a time, whereas men use their bonding capacity to build larger, more hierarchical coalitions(in effect, clubs)^53^. Therefore, it should be also encouraged to enhance the social capital of women with breast cancer in other cultural contexts, thus improve their quality of life.

PROCESS holds great promise for consistent, highly powered, and statistically defensible mediation analyses of indirect effects^54^. Indeed, PROCESS has been heralded in a recent edition of a popular graduate statistics textbook as “pretty much the best thing to happen to moderation and mediation analysis in a long time”^54^. In this study, 5 mediation models were analyzed separately by PROCESS. The advantage is that the consistency and specificity of the effects of different social capital dimensions on QOL can be clarified and compared more intuitively. However, it also has some obvious disadvantages compared with the structural equation model, which is also widely used to analyze mediating effects. PROCESS cannot simultaneously handle the mediation models with latent variables, multiple independent or dependent variables. Therefore, in this study, the mediation analysis with five independent variables cannot be processed in one model, and the results of five mediation models can not be integrated through latent variables in higher dimensions.

The advantage of this paper is that the sample size of this paper has sufficient statistical power if the limitations of cross-sectional mediation are not taken into account. An application developed by Schoemann A M et al. was used to calculate the statistical power of the two serial mediators model^55^. The application was developed based on Monte Carlo Power Analysis Simulation. The correlation matrix of the studies variables was input into the application, and the results showed that the sample size of this study (n = 520) had sufficient statistical power for all five mediation models. For the model of the control over life as independent variable, the statistical power of both two separate mediation is greater than 0.95, while that of serial-multiple mediation is 0.64. In the other four models, the statistical powers of the three mediation paths is greater than 0.90.

The current study had several limitations. Firstly, the results are cross-sectional, which precludes inferring causal relationships. It is not possible to disentangle the temporal relations between these variables and to discern the different directions of the relations. Previous studies have found that cross-sectional approaches to mediation can generate biased estimates of longitudinal parameters, potentially over- or underestimating the effect of the predictor variables on the outcome variable^56,57^. So our findings should be cautiously interpreted. A literature proposed a delineation of mediation effects derived from cross-sectional designs into the terms temporal and atemporal associations to emphasize time in conceptualizing process models^58^. Based on this proposal, this cross-sectional study provides the modest evidence that physical activity and perceived stress could atemporally influence the relationship between individual social capital and QOL among BCSs. Although this study cannot infer the direction of the relationship between variables, the direction that individual social capital precedes physical activity and perceived stress, and that the mediators precede QOL is supported by existing evidences^9,19–21,44,45^. Using mediation analysis as a procedure for delineating the associations, the current cross-sectional mediation analysis could provide initial information as a basis for the more resource-intensive longitudinal studies that can identify the unfolding of the relationships with time. Besides, of practical significance, this study can provide a preliminary framework for QOL interventions in BCSs. Secondly, only two possible mediation variables were included in the mediation analysis model. Other health behavior-related variables such as the diet (e.g. vegetable and fruit intake, diet quality)^9,11^, other psycho-social process-related variables such as anxiety and depression^9,21^, and access to material resources or health services^9^ could be further investigated. Thirdly, this study did not stratify BCSs by their clinical aspects, such as disease stage (early or advanced, etc.) and treatment style (chemotherapy, hormone therapy, radiation, etc.), which should be paid close attention to in the future research. Finally, the current study used self-reported measures and therefore response biases were unavoidable. Regardless of these limitations, this study provides new insight into the relationship between individual social capital and QOL in BCSs using a theoretical exploratory approach. More studies on the mediation of physical activity and perceived stress need to be conducted among cancer survivors.

## Conclusion

This study suggests that in the practice of individual social capital intervention on QOL of BCSs, the constraints which restrain the improvement of physical activity and the reduction of stress level should be identified and eliminated, while individual social capital being enhanced. A multidisciplinary team approach and a variety of delivery systems are needed to address the social, physical and psychological issues for improving QOL among BCSs.

## Data Availability

The datasets generated during and/or analyzed during the current study are available from the corresponding author on reasonable request.
